# Der Telenotarzt als „Einsatzleiter“ zur Stabilisierung eines kritisch kranken Patienten

**DOI:** 10.1007/s10049-021-00945-2

**Published:** 2021-09-23

**Authors:** Dennis Humburg, Maximilian Timpe, Erich Wranze-Bielefeld, Florian Martens, Dennis Rupp, Martin C. Sassen

**Affiliations:** 1 Zentrum für Notfallmedizin, Universitätsklinikum Gießen & Marburg, Standort Marburg, Baldingerstr., 35043 Marburg, Deutschland; 2 Johanniter-Unfallhilfe e. V. Regionalverband Mittelhessen, Linden, Deutschland; 3 Amt für Gefahrenabwehr Vogelsbergkreis, Lauterbach, Deutschland; 4 Klinik für Anästhesiologie, operative Intensivmedizin und Schmerztherapie, Universitätsklinikum Gießen und Marburg, Standort Gießen / Gefahrenabwehr Landkreis Gießen, Gießen, Deutschland; 5 Deutsches Rotes Kreuz Rettungsdienst Mittelhessen gGmbH, Marburg, Deutschland; 6 Zentrum für Notfallmedizin, Universitätsklinikum Gießen und Marburg, Standort Marburg / Fachbereich Gefahrenabwehr Landkreis Marburg-Biedenkopf, Marburg, Deutschland

**Keywords:** Sepsis, Telemedizin, Notfallmedizin, Rettungswagen, Präklinische Versorgung, Sepsis, Telemedicine, Emergency medicine, Emergency mobile units, Emergency medical services

## Abstract

Es wird über die präklinische Versorgung eines 82-jährigen Patienten im septischen Schock bei akuter Pankreatitis berichtet. Initial wurde nur ein Rettungswagen alarmiert, dessen Besatzung nach der ersten Einschätzung einen Notarzt per Hubschrauber an die Einsatzstelle nachforderte. Die Besatzung entschied sich, bei absehbar langem notarztfreien Intervall, telemedizinische Unterstützung anzufordern. Trotz initialer massiver Zentralisierung und schwierigen Bedingungen vor Ort gelang es dem Team, in ständiger Rücksprache mit dem Telenotarzt, den Patienten bis zum Eintreffen des Hubschraubers so zu stabilisieren, dass ein sofortiger und komplikationsloser Transport möglich wurde.

## Abstract (en)

We report about the prehospital treatment of an 82-year-old man with septic shock as a complication of acute pancreatitis. Initially only an advanced life support ambulance was on scene. After the primary assessment, the team decided to call for an emergency-physician-staffed helicopter. Because of the long estimated time of arrival on scene, the paramedics had to stabilize the patient without the physician. Therefore, they requested telemetric support. Despite massive centralization and difficult circumstances, the team succeeded to stabilize the patient in ongoing consultation of the tele-emergency physician. This cooperation made immediate und uneventful transportation after arrival of the helicopter possible.

Wenn eine RTW-Besatzung ein langes notarztfreies Intervall zu überbrücken hat und „load and go“ keine Option ist, kann der Telenotarzt (TNA) wertvolle Unterstützung leisten, indem er den Überblick behält und das Team vor Ort koordiniert.

## Anamnese

Am Abend eines Weihnachtsfeiertags wird ein Rettungswagen (RTW) aus dem mittelhessischen Vogelsbergkreis in einen ländlichen Ortsteil einer Kleinstadt im benachbarten Landkreis Gießen alarmiert. Der RTW ist mit einem Rettungsassistenten (RA) und einer Rettungsassistentin (RAin) besetzt (in Hessen ist bis zum Jahr 2024 noch der Einsatz von RA als Transportführer eines RTW unter bestimmten Voraussetzungen zulässig [5]). Das Meldebild lautet R0 (Notfalleinsatz ohne Sonderrechte) – „Harnverhalt“.

Am Vorabend hatte die Ehefrau des Patienten bereits den ärztlichen Bereitschaftsdienst kontaktiert. Der Patient klagte über seit 3 Tagen progrediente Rückenschmerzen und ein allgemeines Krankheitsgefühl. Zudem habe er in den 24 h zuvor nur sehr wenig Urin ausgeschieden. Der Bereitschaftsdienstarzt stellte bei einem Hausbesuch keine akut behandlungsbedürftige Erkrankung fest und empfahl eine Weiterbehandlung durch den Hausarzt am nächsten Werktag. Heute habe sich dann der Allgemeinzustand weiter verschlechtert und die Urinausscheidung sistierte vollends.

Bekannte Vorerkrankungen sind persistierendes Vorhofflimmern (Antikoagulation mit Dabigatran), leicht reduzierte linksventrikuläre Funktion, Hypothyreose, arterieller Hypertonus und eine im Jahr 2015 festgestellte Koronarsklerose, seinerzeit ohne höhergradige Stenosen.

## Befund

Die RTW-Besatzung wird von der sehr besorgten und aufgeregten Ehefrau erwartet. Sie führt die Kollegen über steile, enge Treppen in das Obergeschoss des Wohnhauses. Dort finden die RA den Herrn auf einem Sofa sitzend vor. Sein Zustand ist zur Überraschung der Kollegen bereits beim Betreten des Raums als kritisch einzustufen. Der Patient ist deutlich tachypnoeisch, kaltschweißig und zeigt ein gräulich-blasses Hautkolorit. Es ergeben sich die in Tab. 1 erhobenen Befunde.

**Table 1:** 

*A* (Atemweg)	Frei Schleimhäute blass und trocken
*B* (Atmung)	Af 50/min S_p_O_2_ 82 % bei Raumluft Thoraxexkursion seitengleich Auskultatorisch beidseits basal exspiratorisches Giemen Ruhedyspnoe wird verneint
*C* (Kreislauf)	Radialispulse nicht tastbar RKZ etwa 8s Extremitäten kalt und marmoriert Massive Kaltschweißigkeit Tachyarrhythmie mit Hf 125/min
*D* (neurologischer Status)	Wach, Augen geöffnet Verwirrt und unscharf orientiert Kein fokal-neurologisches Defizit erkennbar Pupillen mittelweit und isokor
*E* (Einflüsse, erweiterte Untersuchung)	Temperatur 36,0 °C aurikular gemessen Nierenlager beidseits klopfschmerzhaft

*Af* Atemfrequenz, *S*_*p*_*O*_*2*_ peripher gemessene Sauerstoffsättigung, *RKZ* Rekapillarisierungszeit, *Hf* Herzfrequenz

## Arbeitsdiagnose und Nachforderung

Wegen des kritischen Gesamteindrucks und der Vorgeschichte stellen die RA die Arbeitsdiagnose „septischer Schock“ und fordern telefonisch einen Notarzt (NA) nach. Da die beiden nächstgelegenen Notarzteinsatzfahrzeuge (NEF) gebunden sind, entsendet die Leitstelle den Intensivtransporthubschrauber (ITH) „Christoph Gießen“, der im „Dual-use“-Verfahren rund um die Uhr auch für Primäreinsätze genutzt wird.

Da dem RA klar ist, dass es bei Dunkelheit noch wenigstens 20 min dauern wird, bis der ITH eintrifft, fordert er im selben Telefonat bei der Leitstelle Unterstützung durch den TNA an (**Infobox ****1**).

### Infobox 1 Telemedizin im Rettungsdienst in Mittelhessen

Die 3 Landkreise Gießen, Marburg-Biedenkopf und Vogelsberg betreiben seit dem Jahr 2019 das Projekt „Telemedizin im Rettungsdienst in Mittelhessen“. Hierzu sind aktuell insgesamt 15 Rettungswagen (RTW) mit telemetriefähigem Corpuls c3-Monitor/Defibrillatoreinheiten (Fa. GS Elektromedizinische Geräte G. Stemple GmbH, Kaufering, Deutschland) ausgestattet. Die jeweils zuständige Leitstelle stellt nach Anforderung von der Einsatzstelle die Telefonverbindung zum diensthabenden Telenotarzt (TNA) her. Bei den TNA handelt es sich um erfahrene Notärzte aus den beteiligten Landkreisen, von denen sich zu jeder Zeit 2 Kollegen in Rufbereitschaft befinden. Sie sind zur Übermittlung der Monitordaten und insbesondere auch zur Übertragung und Auswertung von EKG mit mobilen Tablet-PC ausgestattet. Somit ist der Dienst von jedem Ort aus möglich, an dem eine WLAN- oder Mobilfunkabdeckung besteht.

Gegenwärtig befindet sich das Projekt noch in einem durch das Hessische Ministerium für Soziales und Integration geförderten Erprobungsverfahren. Daher bestehen in technischer Hinsicht Abweichungen zu den Strukturempfehlungen der Leitlinie „Telemedizin in der prähospitalen Notfallmedizin“ aus dem Jahr 2015 [10]. So wird eine Redundanz auf Seiten des TNA nicht durch technische Voraussetzungen sichergestellt, sondern durch ein jederzeit erreichbares und äquivalent ausgestattetes „Backup“. Auch wird bislang noch auf Bild- bzw. Videoübertragung verzichtet.

Parallel zur Herstellung der Verbindung zwischen RTW und TNA alarmiert der Disponent die örtliche freiwillige Feuerwehr zum Ausleuchten der Landestelle für den ITH und zum Transport des NA an die Einsatzstelle.

Die RAin bietet dem Patienten 8l/min Sauerstoff über Maske an und vervollständigt das Monitoring um endtidale Kapnometrie und 12-Kanal-EKG. Die Messung des Blutdrucks ist zu diesem Zeitpunkt nicht möglich. Aufgrund des B‑Problems (Tab. 1) und der räumlichen Gegebenheiten wird entschieden, den Patienten trotz Schocksymptomatik in sitzender Position zu belassen.

## Diagnose

Der TNA nimmt zuhause den Anruf entgegen, startet seinen Tablet-PC zum Datenempfang und erhält parallel seitens des RA eine strukturierte Übergabe. Der TNA bestätigt daraufhin die Arbeitsdiagnose eines septischen Schocks, am ehesten bei Urosepsis. Differenzialdiagnostisch werden ein anderer Sepsisfokus und die Coronaviruserkrankung 2019 (COVID-19) genannt. Zu diesem Zeitpunkt sind das Tragen von FFP2-Maske und Schutzbrille in jedem Einsatz obligat, sodass sich aus der letztgenannten Differenzialdiagnose zunächst keine Konsequenzen in Hinblick auf den Eigenschutz ergeben.

## Therapie und Verlauf

Gemeinsam werden nun die Abläufe der nächsten Minuten und die Aufgabenverteilung geplant (Tab. 2).

**Table 2:** 

	RA (Position 1)	RAin (Position 2)	TNA
1	Ein Versuch periphervenöser Zugang	Sauerstoff-Flow auf 15l/min erhöhen	Anforderung von Feuerwehrkräften zur Einsatzstelle
2	Wenn 1 frustran, intraossärer Zugang nach Lokalanästhesie	Anbringen Defibrillationselektroden	Beurteilung 12-Kanal-EKG
3	Volumentherapie mittels VEL	Holen und Vorbereiten der Spritzenpumpe aus dem Rettungswagen	–
4	Spritzenpumpe mit Epinephrin starten	Vorbereitung Patiententransport	–
Kontinuierlich	–	Unterstützung der Position 1	Überwachung der Vitalparameter

*RA* Rettungsassistent, *RAin* Rettungsassistentin, *TNA* Telenotarzt, *VEL* Vollelektrolytlösung,

Wegen des kritischen Zustands werden Defibrillationselektroden angebracht und der Sauerstofffluss auf 15l/min erhöht, obwohl die peripher gemessene Sauerstoffsättigung (S_p_O_2_) mittlerweile über 90 % liegt. Der RA hat sich zuvor bereits einen Überblick über die Venenverhältnisse gemacht. Aufgrund der Zentralisation bespricht er mit dem TNA, nur einen Versuch einer Venenpunktion zu unternehmen. Sollte dieser frustran verlaufen, wird die Anlage eines intraossären (i. o.-)Zugangs angestrebt. Priorität hat zu diesem Zeitpunkt die Volumentherapie mittels balancierter VEL. Die parallele Katecholamintherapie mittels Epinephrin über eine Spritzenpumpe (Perfusor®, B. Braun SE, Melsungen, Deutschland) wird im nächsten Schritt geplant. Dass die leitliniengerechte Therapie Norepinephrin vorsieht, dies aber nicht verfügbar ist, wird besprochen. Da auch der TNA aufgrund der Gegebenheiten von einer langen Wartezeit auf den NA ausgeht, wird auch schon der Transport des Patienten in den RTW und eine mögliche Fahrt zum Landeplatz angesprochen.

Eine nichtinvasive Ventilation wird diskutiert, bei aktuell führendem C‑Problem (Tab. 1) aber hintenangestellt. Hinzu kommt die nicht auszuschließende COVID-19-Infektion, wegen der Maßnahmen mit potenziell großer Aerosolfreisetzung zu diesem Zeitpunkt einer sehr strengen Indikationsstellung bedürfen.

Während der nächsten Minuten bleiben TNA und RA in kontinuierlichem Sprachkontakt, sodass jeder Schritt an der Einsatzstelle dem TNA bekannt ist und dieser RA und RAin durch Koordination der besprochenen Schritte und Überwachung der Vitalparameter unterstützt. Der TNA ruft über ein anderes Telefon die Leitstelle an, um über diese einen Teil der Feuerwehrkräfte zur Tragehilfe an die Einsatzstelle zu dirigieren, während der Rest die Ausleuchtung des Landeplatzes übernimmt.

Das dem TNA übertragene EKG zeigt ein tachykardes Vorhofflimmern mit einer Herzfrequenz (Hf) von 125/min, einem Rechts- und einem S1Q3-Typ und einem RS-Umschlag zwischen V3 und V4. Erregungsrückbildungsstörungen sind nicht zu sehen.

Der RA entschließt sich zur i. o.-Punktion an der linken Tibiavorderkante

Da der RA an beiden Armen keine sicher punktierbare Vene entdeckt, entschließt er sich direkt zur i. o.-Punktion an der linken Tibiavorderkante. Nach Hinweis durch den TNA wird zunächst die Punktionsstelle mit 2 ml Lidocain (2 %) unterspritzt. Die Punktion gelingt problemlos und ohne Schmerzäußerung des Patienten. Die restlichen 3 ml Lidocainlösung werden zum Anspülen des intraossären Zugangs mitgenutzt. Die vorbereitete Infusion wird mittels Druckmanschette mit höchstmöglicher Geschwindigkeit („im Schuss“) infundiert.

Im nächsten Schritt bereitet der RA eine Spritzenpumpenspritze mit 3 ml (= 3 mg Epinephrin) ad 47 ml NaCl 0,9 % gemäß Landkreisstandard vor und schließt diese per 3‑Wege-Hahn an den i. o.-Zugang an. Der TNA weist den Start der Spritzenpumpe mit 4 ml/h (4 µg/min) und einem Bolus von 0,5 ml, bei weiterhin fehlendem Radialispuls, an.

Der Patient wird nun für den Transport in den RTW mittels Tragetuch vorbereitet, während Feuerwehrkräfte an der Einsatzstelle eintreffen.

Eine Reevaluation ergibt eine nachlassende Marmorierung der Haut und eine mit etwa 4 s nur noch diskret verlängerte Rekapillarisierungszeit (RKZ). Radialispulse sind aber weiterhin nicht tastbar und eine Blutdruckmessung unmöglich, sodass die Laufrate der Spritzenpumpe auf Anweisung des TNA auf 8 ml/h erhöht wird. Außerdem wird die soeben durchgelaufene Infusion durch eine zweite, ebenfalls „im Schuss“ einlaufend, ersetzt. Der ITH ist deutlich hörbar in der Nähe gelandet, sodass entschieden wird, die Ankunft des NA an der Einsatzstelle abzuwarten.

Da weiterhin kein Blutdruck messbar ist, schlägt der RA nun vor, zur raschen Blutdrucksteigerung zusätzlich Cafedrin und Theodrenalin (Akrinor®, ratiopharm GmbH, Ulm, Deutschland) zu applizieren, was im Notfallrucksack des RTW vorgehalten wird. Der TNA stimmt dem zu und weist die Applikation von 1 ml (100 mg Cafedrinhydrochlorid und 5 mg Theodrenalinhydrochlorid) an. Kurz darauf kann ein erster Blutdruck mit 90/60 mm Hg gemessen werden. Auf Vorschlag des TNA unternimmt der RA nun bei deutlicher klinischer Besserung noch einen Versuch, einen peripheren Venenzugang zu etablieren. Es gelingt die Anlage eines Katheters (16 G) in der rechten Ellenbeuge. Nach einer vollständigen Blutentnahme wird die nunmehr dritte Infusion á 500 ml VEL angeschlossen. Wenige Sekunden später kommt der NA des ITH hinzu. Der Blutdruck kann nun mit 105/68 mm Hg gemessen werden. Die Hf liegt zwischen 90 und 100/min, die S_p_O_2_ um 98 %. Der RA macht eine ausführliche Übergabe nach ABCDE-Schema, die vom TNA mitangehört wird. Da er nichts zu ergänzen hat, verabschiedet sich dieser und beendet Telefonat und Datenübertragung auf sein Tablet.

Der Patient wurde mit Verdachtsdiagnose Urosepsis in ein Universitätsklinikum transportiert

**Figure 1:**
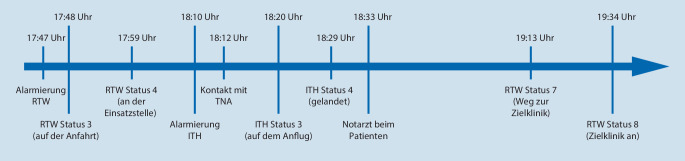

Der Patient wird bodengebunden in Begleitung des ITH-NA mit der Verdachtsdiagnose „Urosepsis“, aber auch mit dem Hinweis „COVID-19 nicht auszuschließen“, zum 29 km entfernten Standort Gießen des Universitätsklinikums Gießen und Marburg (UKGM) transportiert (Abb. 1). Die kontinuierliche Therapie mit Epinephrin wird fortgesetzt. Wegen der guten Wirksamkeit erfolgt kein Umstieg auf das leitliniengerechte Norepinephrin. Der Transport gestaltet sich komplikationslos und der Patient kann in der zentralen Notaufnahme (ZNA) der Zielklinik normotensiv und in deutlich verbessertem klinischen Zustand übergeben werden. Während der Fahrt erhält der Patient weitere 1500 ml VEL, sodass präklinisch insgesamt 3000 ml intravenös infundiert wurden.

Die Bildgebung zeigt eine akute nekrotisierende Pankreatitis, chronische Rechtsherzinsuffizienz und eine Leberzirrhose mit Aszites. Die Laborkonstellation weist darüber hinaus deutlich auf eine Sepsis hin.

Epinephrin kann zunächst reduziert und der Patient nach COVID-19-Ausschluss auf eine Intermediate-Care-Station verlegt werden. Eine Antibiose mit Meropenem wird initialisiert.

Nach anfänglicher klinischer Besserung steigen am Folgetag Infektwerte und Katecholaminbedarf. Es erfolgt die Verlegung auf die Intensivstation. Wiederum einen Tag später wird der Patient bei respiratorischer Insuffizienz intubiert und verstirbt trotzdem 3 Tage nach stationärer Aufnahme.

## Diskussion

Leider konnte das Ineinandergreifen der präklinischen Ressourcen hier nicht zu einem positiven Outcome beitragen. Mit einem direkt alarmierten NEF wäre der Einsatz sicherlich schneller, die Therapie aber mutmaßlich gleich verlaufen – mit einer Ausnahme: Die Verwendung von Epinephrin als erster Vasopressor widerspricht der aktuellen S3-Leitlinie zur Behandlung der Sepsis [3]. Norepinephrin wird momentan nicht auf den RTW in den mittelhessischen Landkreisen vorgehalten. Der regelmäßige Einsatz der Telemedizin bei kritisch kranken Patienten gibt sicherlich Anlass, dies nun zu überdenken.

Eine Verzögerung von Versorgung oder Transport durch den TNA kann sicher ausgeschlossen werden

Eine Verzögerung von Versorgung oder Transport durch den Einsatz des TNA kann hier sicher ausgeschlossen werden. Die vor Eintreffen des NA getroffenen Maßnahmen waren zwingend erforderlich, um überhaupt Transportfähigkeit herzustellen.

Vielmehr zeigt dieser Einsatz, wie mithilfe der Ressource TNA das notarztfreie Intervall bei kritisch kranken Patienten sinnvoll überbrückt werden kann. Zweifelsohne hätte die Mehrzahl der getroffenen Maßnahmen vom Team des RTW auch ohne Zuhilfenahme der Telemedizin getroffen werden können. Lediglich die Applikation von Cafedrin und Theodrenalin gehört in den mittelhessischen Landkreisen nicht zu den „erweiterten Versorgungsmaßnahmen“ (EVM), die Notfallsanitäter und RA unter gewissen Umständen eigenverantwortlich durchführen. Die Nutzung einer Epinephrin-Spritzenpumpe ist lediglich bei bedrohlicher Bradykardie und Anaphylaxie Teil der EVM [2].

Ob im konkreten Fall tatsächlich die β_1_-mimetische Wirkung (Inotropie) des Theodrenalins den Ausschlag dafür gab, dass bei bereits wieder tastbaren Pulsen nun auch ein Blutdruck messbar wurde, bleibt unklar. Es ist ebenso denkbar, dass dieser Effekt auf der Bolusgabe von 1 ml Volumen über den, zu diesem Zeitpunkt, einzigen venösen Zugang beruhte, an dem auch das Epinephrin einlief. In diesem Fall hätte eine weitere Bolusgabe bzw. Laufratenerhöhung des Katecholamins den gleichen β_1_-Effekt mit zusätzlicher stärkerer Wirkung auf α_1_-Rezeptoren (Vasokonstriktion) gezeigt [1].

Die Anlage eines intraossären Zugangs gehört selbstverständlich zum Standardrepertoire des Rettungsfachpersonals. Allerdings wird sie nur selten beim wachen Patienten durchgeführt. So wurde die Lokalanästhesie der Punktionsstelle in diesem Fall erst auf Hinweis des TNA durchgeführt, so wie es die entsprechende Leitlinie vorsieht [4]. Bei aller Standardisierung und allem Training sind auch für erfahrene Rettungsdienstmitarbeiter die Maßnahmen jedenfalls nicht alltäglich.

Berücksichtigt werden muss aber auch, dass diese komplexe Einsatzsituation nicht nur aus dem Patientenzustand resultierte. Vielmehr kamen hier schwierige räumliche Verhältnisse, wodurch gerade die RAin des RTW mehrfach mehrere Minuten gebunden war, und weitere einsatzlogistische Herausforderungen hinzu. Dies unterstreichen auch die langen Latenzzeiten zwischen Eintreffen des RTW am Wohnhaus und Nachforderung des Notarztes bzw. Ankunft des NA und Abfahrt des RTW mit dem Patienten (Abb. 1). Auch die Betreuung des Patienten und der aufgeregten Angehörigen im Hinblick auf die invasiven Maßnahmen (intraossärer Zugang am wachen Patienten) forderte dem RTW-Team mentale Ressourcen ab.

Die Aufgabe des TNA war kaum mit der eines „normalen“ Notarztes vergleichbar

Insofern war die Aufgabe des TNA hier kaum mit der eines „normalen“ Notarztes vergleichbar. Schon im herkömmlichen Telemedizineinsatz liegt es in der Natur der Sache, dass der TNA nicht selbst die Untersuchung und Behandlung vornimmt. Vielmehr analysiert er die ihm vorliegenden Informationen und delegiert die Durchführung der Maßnahmen. Umso mehr gilt dies bei einem komplexen Einsatzgeschehen, dass mit einer Vielzahl an Informationen, aber auch notwendigen Maßnahmen durch verschiedene Teammitglieder einhergeht. Hinzu kommt die Möglichkeit, die Kommunikation „nach außen“, beispielsweise mit der Leitstelle, durch den TNA zu übernehmen. Nach Ansicht der Autoren bestehen somit große Überschneidungen zwischen den Anforderungen an den TNA in einem solchen Geschehen und einer Tätigkeit als Einsatzleiter bei größeren Schadenslagen oder auch als „Schockraum-Leader“. Nicht zuletzt aufgrund dieser „Einsatzleiter“-Aufgaben ist für die Mitarbeit als TNA im Projekt der Autoren eine aktive Tätigkeit als leitender Notarzt oder alternativ eine leitende Funktion in einer zentralen Notaufnahme Voraussetzung.

Wie in *Infobox **1* zum Projekt beschrieben wird aktuell eine Videoübertragung von der Einsatzstelle zum TNA nicht (bzw. nur in einem frühen Versuchsstadium) genutzt. Nach Überzeugung der Projektverantwortlichen ist diese auch in der absoluten Mehrzahl der Telemetrieeinsätze verzichtbar. Dies bestätigen ebenfalls die nach jedem Einsatz durchgeführten Evaluationen durch die RTW-Besatzungen und die TNA. In komplexen Einsatzgeschehen bei kritisch kranken Patienten aber, wie also im hier berichteten Fall, wäre eine Bildübertragung nicht nur zur Einschätzung des Patientenzustands hilfreich. Vielmehr wäre es so dem TNA möglich, ohne verbales Feedback jederzeit zu erkennen, welches Teammitglied gerade welche Maßnahme durchführt bzw. durchgeführt hat und für die nächste Aufgabe zur Verfügung steht. Diese Möglichkeit fehlte hier, ist aber auch nicht mit in den RTW fest verbauten Kameras gegeben.

Die Übernahme der Einsatzleitung durch den TNA entlastet maßgeblich den RA am Einsatzort

Trotzdem entlastete die Übernahme der Einsatzleitung durch den TNA maßgeblich den RA an Position 1 an der Einsatzstelle, wodurch dieser sich auf die korrekte Durchführung invasiver Maßnahmen und die Patientenbeobachtung konzentrieren konnte. Entscheidend war in der konkreten Einsatzsituation sicherlich, dass sich das Team gezielt Werkzeugen des „Crew Resource Management“ wie „Closed Loop Communication“ (Fehlervermeidung durch Rückbestätigung) und eines „10 for 10“ („10 s für 10 min“) zu raschen Planung der Abläufe der nächsten Minuten bediente [6, 8]. Die Tatsache, dass RA und TNA zuvor schon häufig gemeinsam als Simulationstrainer (Notarzt- und Schockraumtrainings etc.) tätig waren, war hier sicher hilfreich.

Kritisch anzumerken ist rückblickend, dass keine vollständige körperliche Untersuchung des Patienten erfolgte. Bis zum Eintreffen des ITH-Notarztes musste diese zur Stabilisierung der Vitalfunktionen zurückstehen. Anschließend erfolgt hätte sie möglicherweise den richtigen Sepsisfokus früher zum Vorschein gebracht, mutmaßlich aber ohne Auswirkungen auf die präklinische Therapie. Eine strukturierte Übergabe, z. B. nach dem ISOBAR- oder SBAR-Schema, die gezielt auch die noch ausstehenden Maßnahmen beinhalten, hätte diesem vielleicht vorgebeugt [11].

Schließlich sind auch 2 Aspekte diskussionswürdig, die außerhalb des eigentlichen präklinischen Einsatzgeschehens liegen: Der Besatzung war innerhalb weniger Augenblicke klar, dass sie es mit einem kritisch erkrankten Patienten zu tun hatte. Es bleibt unklar, warum dies bei der Notrufabfrage nicht erkannt wurde, was dann zu einem rascheren Eintreffen von RTW und Notarzt geführt hätte. Denkbar ist, dass dies mittels „strukturierter Notrufabfrage“ (SNA), die im Rettungsdienstbereich Gießen erst im kommenden Jahr eingeführt werden soll, besser gelungen wäre. Allerdings liegt es ja gerade in der Natur des „notfallmedizinischen Chamäleons“ Sepsis, dass es sich an unspezifischen Störungen verschiedener Organsysteme zeigt. Möglicherweise hätte das Warnzeichen „Kaltschweißigkeit“ bei konsequenter Anwendung der SNA eine höhere Dringlichkeit ergeben. Sicher erscheint dies aber nicht, zumal der Patient selbst sich subjektiv gar nicht besonders symptomgeplagt zeigte. Auch die Übergabe des Patienten in der ZNA ist zu hinterfragen. Mittlerweile steht die Empfehlung zur Versorgung kritisch kranker konservativer Patienten in einem nichttraumatologischen Schockraum auf breiter Basis [7, 9]. Am Standort Gießen des UKGM erfolgt dies nicht innerhalb der ZNA, sondern auf einer Intensivstation. Eventuell muss dieser vorgesehene Ablauf deutlicher kommuniziert oder überdacht werden. Auch bei diesem Punkt ist es natürlich höchst spekulativ, ob die Versorgung in einem Schockraum zu einem besseren Outcome beigetragen hätte.

## Fazit für die Praxis

- Ein Telenotarzt (TNA) kann nicht selbst aktiv am Patienten handeln. Allerdings kann er durch Reduktion der Aufgabenlast helfen, auch die Durchführung seltener Maßnahmen für das Team an der Einsatzstelle deutlich zu erleichtern. Gut ausgebildetes Rettungsdienstpersonal ist die Voraussetzung.
- Telemedizin im Rettungsdienst kann gerade auch beim kritisch kranken Patienten, schwierigen räumlichen Verhältnissen und komplexem Einsatzgeschehen helfen, die Übersicht über die getroffenen und noch zu treffenden Maßnahmen zu behalten (TNA als „Einsatzleiter“).
- Zur Erleichterung dieser „Einsatzleiter“-Tätigkeit bei komplexen Einsätzen ist eine Videoübertragung von der Einsatzstelle zum TNA unerlässlich, auch wenn diese bei der Mehrzahl der Telemedizineinsätze keine Rolle spielt.
